# The Formation and Decay of an Unstable State of a Suspension of Hydrophobic Nanoporous Particles under Rapid Compression

**DOI:** 10.3390/nano11010102

**Published:** 2021-01-04

**Authors:** Vladimir Borman, Anton Belogorlov, Vladimir Tronin

**Affiliations:** 1 Polymeric Membranes Laboratory, A.V. Topchiev Institute of Petrochemical Synthesis, Russian Academy of Sciences, Leninsky Prospekt, 29, 119991 Moscow, Russia; VDBorman@mephi.ru; 2 Molecular Physics Department, National Research Nuclear University MEPhI, Kashirskoe sh. 31, 115409 Moscow, Russia; VNTronin@mephi.ru

**Keywords:** porous medium, non-wetting liquid, impact, metastable state

## Abstract

The study of non-wetting liquid transport in a nanoporous medium is stimulated by the possible use of this process to absorb or accumulate mechanical energy. The filling of nanopores of suspended particles with a non-wetting liquid under decay of the unstable state, when the pressure increase rate is much higher than the rate of volume change, is studied. Based on the new experimental data and a theoretical model of the interacting modes of the spontaneous filling and filling under rapid compression, a picture of the percolation transition and a mechanism of liquid transport under such conditions are proposed. It is shown that a new dynamic filling threshold $P_{0}$ is reached. It is shown that the filling of the porous medium is the result of the slow mode of impact compression when the fast mode of spontaneous filling is continuously adjusted to the slow mode on a small time scale. The theoretical model of the interacting modes is based on the solving of a system of kinetic equations for the distribution functions f(n,t) and F(n,t) clusters of filled pores under rapid compression, respectively. It is shown that filling at P=const corresponds to the non-dissipative transport of liquid on a time scale smaller than the characteristic filling time. The proposed model quantitatively describes the experimental data. So, the response of suspension to impact is characterized by the positive feedback.

## 1. Introduction

The problem of describing fluid transport in a nanoporous medium, due to the fundamental complexity of the necessary consideration of fluid correlations in pores of different locations and high demand for various applications, has been the focus of attention for many decades [1,2].

More recently, new directions in the study of transport in the zeolites subnanometer channels, in nanometer pores of disordered media such as silica gels, carbon nanotubes, metal-organic structures have arisen [3,4,5,6,7,8]. To describe molecular transport in nano–subnanostructures, it is necessary to take into account local and long-range correlations of liquid in different pores. At the same time, the study of transport allows us to develop models that adequately describe such correlations. The study of non-wetting liquid transport when filling a disordered nanoporous medium is stimulated by the possible use of this process to absorb vibrations, damping, mitigate the impact, and protect against explosion [9,10,11]. Such applications are based on high liquid transport rates. The characteristic time ($\tau_{V}$) of filling nanopores with a radius of R=1−100 nm in microparticles with a size of L=1−100 μm is $10^{-4}-10^{-1}$ s and may be close to the characteristic time of external impact [12,13]. Repeated use is possible if porous media and liquids are used, which, when impacted, are in an unstable state with filled pores, and the liquid quickly flows out with a decrease in pressure [13]. In the intrusion—extrusion regime close to quasistatic filling, the dependencies of pressures $P_{int}$ and $P_{ext}$ for zeolites [3], for silica gels with a modification to provide hydrophobic properties to the surface of pores and non-wetting liquids—water, aqueous solutions of salts and organic substances, were determined [14,15,16,17]. It was also established that the filling pressure $P_{int}$ for various systems increases with an increase in the transport velocity of non-wetting liquids in porous microparticles [18]. This is observed in a regime close to quasistatic filling, when the characteristic time of elastic compression is $\tau_{p}∼\tau_{V}$. Experiments with various viscous liquids showed that this dependence of the pressure $P_{int}$ can be associated with viscous friction losses with an increase in the transport velocity when the liquid slips on the pore walls [18]. According to [7], the effective viscosity decreases with increasing liquid flow rate.

To date, several studies [10,11] of the process of filling nanopores with rapid compression in dynamic mode have been performed. Suspensions with hydrophobic microparticles of silica gels, zeolites in water, and aqueous solutions of salts were studied. Shock compression techniques were used when a load shock of 1÷100 ms drives a rod entering a chamber with a compressible suspension [10,11]. The Hopkinson Bar or Split Hopkinson Bar methods were also used, when the suspension was placed in the chamber between two rods, and hitting one of them led to the propagation and attenuation of the compression wave of the suspension [12,19,20]. From the first works, it followed that for the systems Wood’s liquid alloy–silokhrom SCh-1.5 [21] and silica gel Fluka 100 C8—water [12] the filling upon impact occurs at a pressure exceeding the pressure of quasistatic filling and, therefore, beyond the percolation threshold. Therefore, it can be assumed that with a decrease in the characteristic compression time $\tau_{p}$, the nanopore filling mechanism changes.

A model for describing slow quasistatic filling and fast compression filling was published in [13]. The description of the percolation process as a fictitious dynamic process outlined in the work of Abrikosov was used [22,23]. It was proposed to describe the filling of a nanoporous medium with a non-wetting liquid using the time-dependent distribution functions of clusters of accessible but empty pores and of filled pores. It is assumed that the accessible pores will form without delay, according to the achieved pressure value. It follows from the solution of the system of kinetic equations for these functions that during fast compression, the filling process should occur only after reaching a new threshold in terms of the fraction of accessible pores $\theta_{c}=0.28$. This value is above the quasistatic percolation filling threshold $\theta_{c0}=0.18$ [24].

It follows from the solution [13] that the frequency spectrum of the relaxation of the distribution function of clusters of filled pores consists of a solitary positive low frequency and negative high frequencies, depending on the size of the cluster. These negative frequencies correspond to the relaxation of intrusion-extrusion fluctuations of the liquid from clusters of accessible pores. The solitary frequency of the evolution of the unstable mode does not depend on the size of clusters of filled pores and describes their collective spontaneous growth at the critical pressure. This rapid growth mode should occur when the pressure is greater than the quasistatic pressure of the non-wetting liquid intrusion into the pores. The predicted exponentially accelerated decay of the unstable state of suspension, however, was not observed [10,11,12,19,20,21]. These studies did not measure the time dependence of a volume of filled pores [10,11,12,19,20] and the flux of filling.

In the present work, new experimental results on the formation and decay of the unstable state of the suspension (Section 3) were obtained. A linear time dependence of the normalized flux and a quadratic time dependence of the normalized volume of filled pores have been found. These dependencies correspond to the decrease of liquid transport rate under the pressure, which occurs at impact slowdown. However, these dependencies are observed at a constant pressure, independent of the transport rate. Such situation can correspond to spontaneous non-dissipative transport of non-viscous liquid. Thus, the task of describing the obtained new experimental data appears. The problem of unstable state decay of the nanopore system in a non-wetting liquid has been solved (Section 4 and Section 5). The resulting picture of transport and pore-filling is based on taking into account the interaction of spontaneous transport and transport resulting from impact. Concluding remarks are given in the Section 6.

## 2. Materials and Methods

The main studies were performed for the system granular nanoporous medium—Libersorb 23 (L23) and non-wetting liquid—distilled water (DW). The porous material L23 is KSK-G silica gel with the SiO${}_{2}$ skeleton material, the surface of which was chemically modified with alkylsilanes, to produce hydrophobic properties to the pore surface [25]. Using the methods of low-temperature nitrogen adsorption [26], helium pycnometry [26], and liquid porometry [17], the following characteristics of porous media were determined and within the limits of the error coincide with the results obtained in [27]: specific pore volume ($V_{por}$) (0.56±0.02) cm${}^{3}$/g, skeleton density ($\rho_{pm}$) (1.7798±0.0016) g/cm${}^{3}$, specific surface area ($S_{por}$) (212±7) m${}^{2}$/g (multipoint BET), specific volume of the porous medium $(V_{pm}=1/\rho_{pm}+V_{por})∼1.22$ cm${}^{3}$/g, granule size (*L*) ∼10 μm, average pore size (<R>) (5.0±0.2) nm, porosity (φ) ∼0.52 and characteristic of the pore size distribution function (δR/<R>) ∼0.1. In addition, the compressibility of an unfilled porous medium ($\chi_{pm}$) $∼0.41\cdot 10^{-2}$ MPa${}^{-1}$, the pressure value of the beginning of filling of pores under the conditions of a slow (quasistatic) pressure change ($P_{c0}$) (15±1) MPa, independent of the velocity of the rod according to [17]. The compressibility of the liquid ($\chi_{liq}$) $4.4\cdot 10^{-4}$ MPa${}^{-1}$ according to [28].

In the experiment, 5 g of the porous medium L23 was placed in the permeable for the liquid container in a high-pressure chamber with a volume of ∼60 cm${}^{3}$. The inter-granular space in the container and the remaining volume of the chamber were filled with DW. Through the seal in the chamber the rod with diameter 10 mm was inserted. Studies of the dynamics of filling porous systems with non-wetting liquids were carried out on the experimental bench and according to the method described in [13].

The experimental bench consisted of upper and lower plates, fixed by pipes. Steel ropes were stretched between the plates, over which the load by mass 10 kg could freely slide. A strain gauge force sensor was installed on the bottom plate. The sensor measures a force from 10 to $10^{4}$ N with an error of less than 5% at a force value of more than 100 N. The sealed high-pressure chamber filled with liquid and porous medium with inserted rod was installed on force sensor. The internal volume of the chamber is changed due to the movement of the rod, inserted into the chamber through the seal. The rod of the high-pressure chamber is rigidly connected by a steel plate to the rod of the displacement sensor with a stroke of 15 cm and a measurement error of 0.5%.

In the experiment, time dependencies of a force *F* and chamber rod displacement *l* upon impact of the load were measured. The relationship between the pressure in the chamber and the recorded force acting on the force sensor was determined by the relation P=F/S, where *S* is the cross-section area of the rod, and the change in the internal volume of the chamber was determined as ΔV=lS. The data acquisition rate was 5 kHz. Signals from sensors through the analog-to-digital converter (ADC) were recorded and processed using a computer. The design of the stand made it possible to vary the impact energy from 3 to 100 J. The impact energy was determined as $E=m_{g}gh$. Where $m_{g}$ is the mass of the load, *h* is the distance between upper plain of the chamber rod and the load, *g* is the free-fall acceleration.

When the rod moves inside the chamber, the volume of the suspension decreases. This decrease in volume can be due both to the elastic deformation of the suspension, and to the filling of nanopores of granules with a non-wetting liquid, caused by an increase in pressure during compression. The rod movement (*l*) recorded in the experiments to determine the time dependence of the suspension volume (ΔV) and, therefore, the rate of volume change $J=d\Delta V/dt=v_{r}S$ (where $v_{r}=dl/dt$ is the velocity of the rod). These dependencies, together with the measurement of the dependence of pressure on time (*P*) characterize the effect of impact and the response of the suspension to impact.

**Figure 1:**
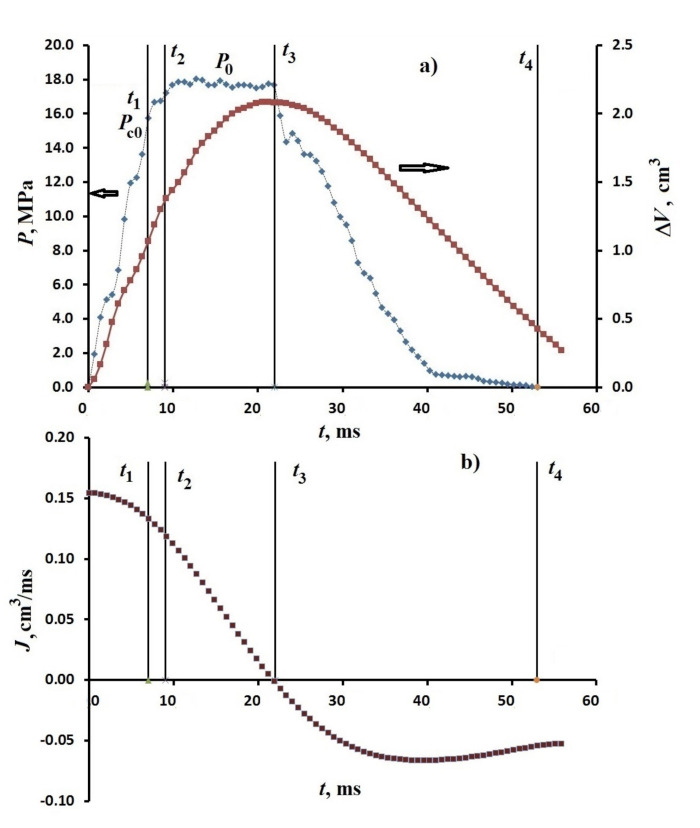
Time dependencies (**a**) of the pressure *P* and volume change ΔV and (**b**) the rate of the volume change *J* for the L23 (5 g)—water system (55 cm${}^{3}$) at the impact energy E= 30 J at 20 ℃.

Figure 1 shows the dependencies *P*, ΔV and *J* for the suspension L23 (m=5 g) – water (V=55 cm${}^{3}$) with impact energy E=30 J (load mass 10 kg, initial velocity of the rod 2.5 m/s). The figure shows the main time points. The time $t_{1}$ corresponds to the time for reaching the characteristic pressure to fill the porous medium with liquid in the quasistatic regime $P_{c0}$, according to [17]. The second point $t_{2}$ is defined as the transition time of the system to spontaneous filling at constant pressure. The time $t_{3}$ is determined as the rod stop time along with the load, i.e., the time of conversion of the mechanical energy of the load with the rod into the internal energy of the system. Time $t_{4}$ corresponds to the time of detach of the load from the rod. It follows from the figure that in the time interval up to $t_{2}$ an increase in pressure and a decrease in the volume of the suspension are observed with a decrease in the velocity of the rod. In the vicinity of the time $t_{2}$, the pressure increase rate (dP/dt) changes abruptly with continuous time dependencies of the volume ΔV and the rate of change in the volume (*J*). Over the time interval from $t_{2}$ to $t_{3}$, the pressure remains constant within the measurement error, and the rate *J* vanishes at the point $t_{3}$. The volume change at the point $t_{3}$ reaches its maximum value. After the point $t_{3}$, the volume and pressure return to their original values. Thus, the response to an impact with a pressure increase that is close to linearly and the volume decrease to the point $t_{2}$ is replaced by a volume change at constant pressure $P=P_{0}=const$, and a decrease in the value of *J* to zero at the point $t_{3}$. The time moment $t_{3}$ corresponds to the stop of the rod and the subsequent return movement. It should be noted the value of $P_{0}$ at the time $t_{2}$, when the derivative dP/dt changes abruptly. The value of $P_{0}$ is greater than the value of pressure $P_{c0}$, at the point $t_{1}$ (see Figure 1) at which filling begins in quasistatic mode. The dependencies *P* and ΔV similar to those discussed above were observed earlier for the other investigated systems [13,27,29].

## 3. Various Modes of Filling Nanopores with Rapid Compression

**Figure 2:**
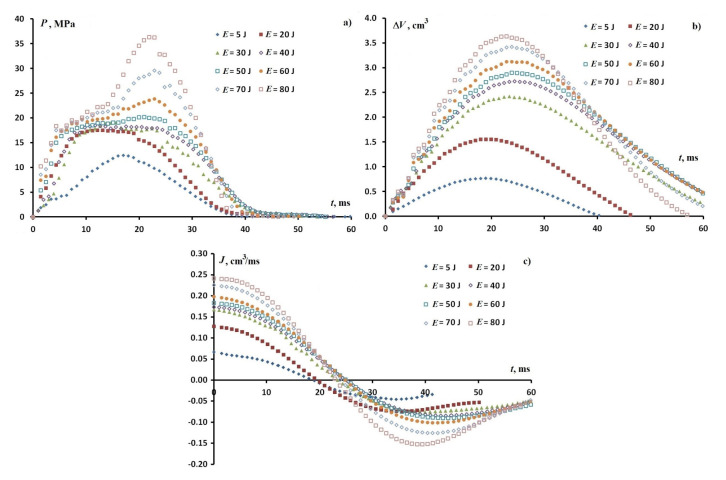
Time dependencies of the (**a**) pressure *P*, (**b**) volume change ΔV, and (**c**) rate of the volume change *J* for the L23 (5 g) – DW (55 cm${}^{3}$) system at impact energies 5, 20, 30, 40, 50, 60 , 70 and 80 J at 20 ${}^{\circ }$C.

Series experiments were carried out to study modes of filling nanopores with rapid compression. Figure 2 shows the dependencies on time, volume change ΔV, rate of the volume change *J* and pressure *P* for the L23–DW suspension at impact energies E=(5÷80) J. In the energy range (20÷50) J the filling pressure remains constant, which corresponds to the linear dependence *J* and is equal to $P_{0}=18$ MPa, and for $P=P_{0}$ a jump of $\Delta \dot{P}$ is observed, and the dependence of volume on time is close to quadratic. As the energy E>50 J increases the pressure *P* increases linearly. At an energy of E=70 J, a second jump of $\Delta \dot{P}$ and a further increase in pressure to a maximum are observed when the volume versus time dependence is close to symmetric, characteristic for elastic compression. The rate of change in the volume of *J* increases and, with an increase in energy to E=70 J, tends to the value characteristic of elastic compression.

From the obtained dependencies in Figure 2 it follows that at an energy of E=(20÷50) J, the response of the suspension can be explained as the process of the irreversible transition of the suspension to a stable state with filled particle pores at constant pressure $P=P_{0}$. This shift of the boundary in energy of the filling region at constant pressure $P=P_{0}$ is associated with the condition that the filling rate of pores *J* is equal to the compression rate $v_{r}S$. From this condition it follows that to maintain the regime $P=P_{0}$ with increasing energy, it is necessary to increase the filling rate. Otherwise, with an increase in the impact energy, the compression rate $v_{r}S$ will exceed the maximum filling rate of the pores of all granules $J^{max}$. In this case, the rate of volume change, as the response of the suspension, will be determined by the sum of the maximum pore-filling rate at P=const and the elastic compression rate $J_{el}$ of the suspension: $v_{r}S\approx J^{max}+J_{el}$. In accordance with this equality and the formula for the relative pressure change rate, $\dot{P}={\tilde{\chi }}^{-1}\Delta \dot{V}$ with elastic compression, the value $\dot{P}$ is equal to $\dot{P}=\frac{v_{r}S-J^{max}}{\tilde{\chi }}$. Here $\tilde{\chi }$ is the dimensionless elastic compressibility of the suspension. This dependence and the results shown in Figure 2 allow an understanding that for E=70 J the jump in the pressure increase rate, $\dot{P}$ is less than for E=50 J, and the pressure dependence on time is close to linear. At a compression rate significantly exceeding the maximum filling rate, if $v_{r}S\gg J^{max}$, the value $\dot{P}$ will be determined by the elasticity of the system. This should be observed either at a high compression rate upon impact, or with a small mass of granules [10,12]. In the mode at P=const, the acceleration of the flow deceleration is constant. This means that the response of the suspension in the regime of irreversible decay of the non-equilibrium state of the suspension determines the impact absorption process. Since the filling slows down at P=const, this means that the hydrodynamic resistance of the pore system of the granules depends on the filling rate *J*.

## 4. Kinetics of the Formation and Decay of an Unstable Suspension State

From the previous section it follows that the process of changing the state of a suspension of particles with empty pores during rapid compression consists of two successive stages—elastic compression, during which the pressure increase rate is higher than the rate of volume decrease, and the subsequent threshold filling of the pores, when the rate of volume decrease due to the filling of pores exceeds the pressure increase rate. Under elastic compression, when the quasistatic filling pressure $P_{c0}$ is exceeded and achieves the value $P_{0}$, the number of accessible pores grows, an infinite percolation cluster of those pores is being formed, and the number of accessible pores in this cluster increases. On the other hand, under elastic compression, until the threshold pressure $P_{0}$ is reached, filling due to the delay is not observed within the measurement error (≤5%), so that at $P<P_{0}$ only separate filled pores and clusters of filled pores are found, if only they are formed at all. Therefore, the response, the temporary evolution of a suspension, during rapid compression can be described as the process of formation during the compression of an unstable state of particles with empty pores in a non-wetting liquid at a threshold pressure of $P_{0}$, the subsequent possible formation of clusters of filled pores during the liquid intrusion time and filling of pore clusters, and then the threshold spontaneous filling of the percolation cluster from empty pores.

In a small neighborhood of the filling threshold, the average cluster size of filled pores is close to the correlation length $\xi =\bar{R}\cdot {\left|\theta -\theta_{c}\right|}^{-\nu }$ ($\bar{R}$ is the average pore size, ν∼0.8 is the critical index [24]), and it becomes comparable with particle size. Therefore, filling can be considered to be a process starting from the surface of particles and proceeding simultaneously in the entire space of connected pores. Then, the task of describing pore-filling can be considered to be the task of calculating the coordinate independent distribution functions of clusters from accessible and filled pores under conditions of rapid compression, when the pressure and fractions of accessible and filled pores depend on time.

In our calculations, it was assumed that the pore radius distribution is narrow, ΔR/R<1, but ΔR≠0, so the percolation transition depends on the spread of pore radii and on the connectivity between pores. Below, when obtaining kinetic equations for the distribution functions of clusters from *n* accessible pores f(n,t) and from *n* filled pores F(n,t) it is assumed that the filling of an accessible pore leads only to the disappearance of the accessible pore and the medium being filled does not change during the filling process. However, when calculating the filled volume, the change in the medium is taken into account in the mean-field approximation.

Cluster formation in the ball problem (white and black balls) was described in [22], where the distribution function of white ball clusters by the number of balls in them was introduced. The change in the distribution function in this model occurs due to the formation of clusters of white balls. Following the work [22], we describe the dynamics of filling granules of a porous medium with a non-wetting liquid, assuming that the medium for filling consists of accessible pores. In this case, the role of white balls is played by accessible pores, and their proportion is determined by the ratio

(1) $$\theta \left(P\left(t\right)\right)=\int_{0}^{\infty }w\left(R,P\left(t\right)\right)f_{r}\left(R\right)R^{3}dR,$$

where $f_{r}\left(R\right)$ is the pore volume distribution function, $w\left(R,P\left(t\right)\right)=w_{0}\exp^{-\delta A/T}$—the probability of filling the pores in accordance with [30]. Here δA is the potential barrier for the intrusion of the liquid into the pore. According to (1), the pores are accessible if they can be filled at a pressure of *P* as a result of fluctuation filling. However, filling can only occur as a result of a kinetic process in a finite time $\tau_{0}∼w_{0}^{-1}$.

In describing the dynamics of filling porous particles with a non-wetting liquid, the pressure and the fraction of pores accessible for filling depend on time. With this in mind, the kinetic equations that determine the time evolution of the distribution functions of the clusters of accessible and filled pores by the number of pores can be written in the form [13]

(2) $$\frac{\partial F\left(n,t\right)}{\partial t}=\sum_{m=1}^{n-1}F\left(m,t\right)\frac{f\left(n-m,t\right)}{\tau \left(m,n-m\right)}-\sum_{m=1}^{\infty }F\left(n,t\right)\frac{f\left(m,t\right)}{\tau \left(n,m\right)}-F\left(n,t\right)\frac{S\left(\varepsilon \left(t\right)\right)}{\tau_{pc}\left(n\right)},$$

(3) $$\begin{array}{c} \frac{\partial f\left(n,t\right)}{\partial t}=\frac{1}{\tau_{d}}\left[\frac{1}{2}\sum_{m=1}^{n-1}m^{q}{\left(n-m\right)}^{q}f\left(m,t\right)f\left(n-m,t\right)-\right.\\ \left.-n^{q}f\left(n,t\right)\sum_{m=1}^{\infty }m^{q}f\left(m,t\right)-2n^{q}f\left(n,t\right)S\left(\varepsilon \right)\right]-\\ -\sum_{m=1}^{n-1}F\left(m,t\right)\frac{f\left(n-m,t\right)}{\tau \left(m,n-m\right)}+\sum_{m=1}^{\infty }F\left(n,t\right)\frac{f\left(m,t\right)}{\tau \left(n,m\right)}+F\left(n,t\right)\frac{S\left(\varepsilon \left(t\right)\right)}{\tau_{pc}}, \end{array}$$

where

(4) $$S\left(\varepsilon \right)=\varepsilon^{\delta }\Theta \left(\theta -\theta_{c0}\right),\;\varepsilon \left(t\right)=\left|\theta \left(t\right)-\theta_{c0}\right|,$$

(5) $$\tau_{d}={\left(\frac{\partial \varepsilon }{\partial t}\right)}^{-1}=\varepsilon^{1+\gamma }\left(t\right)\tau_{p},\;\tau_{p}={\left(\frac{dp}{pdt}\right)}^{-1},$$

where $\tau_{p}$ is the characteristic time of pressure change, $\tau_{pc}$ is the characteristic time of filling an infinite cluster of accessible pores from filled clusters, $\tau_{d}$ has the meaning of the characteristic time of formation of accessible pores when the pressure changes in time, *q*, δ, γ are critical indices, S(ε(t)) is the effective part of an infinite percolation cluster of accessible pores, i.e., the fraction of pores belonging to an infinite cluster through which it can be filled, Θ(x) is the Heaviside function. The existence of a percolation cluster of accessible pores must be taken into account, since it forms at $P=P_{c0}$ and $\theta =\theta_{c0}$.

Equation (2) defines the distribution function of clusters of filled pores at an arbitrary point in time. The first term describes the formation of a cluster of *n* pores as a result of filling clusters of n−m accessible pores through clusters of *m* filled pores in the characteristic time τ(m,n−m). The second term corresponds to joining when filling to a cluster of *n* filled pores of any cluster from accessible pores for the characteristic time τ(n,m). The third term describes the filling of the percolation cluster of accessible pores from the filled clusters in the characteristic time $\tau_{pc}\left(n\right)$. Equation (2) does not take into account changes in the distribution function F(n,t) due to the merging of clusters of filled pores with each other, which corresponds to the assumption that the medium remains constant during the filling process. The function F(n,t) under conditions of almost complete filling is calculated below in the mean-field approximation.

Equation (3) determines the time evolution of the distribution function of the clusters of accessible pores due to their merging with each other (the first two terms), joining an infinite cluster (third term) and the processes of intrusion-extrusion of liquid from them (last three terms). Depending on the characteristic times τ(n,m) and $\tau_{pc}\left(n\right)$ in (2) and (3) on the number of pores can be obtained from the following considerations. For estimation, the volume of a *V* cluster of *m* pores with the same radii $\bar{R}$ is $V=\frac{4}{3}\pi {\bar{R}}^{3}m$, the area *s* of the contact of two interacting clusters of *m* and *n* pores then is equal to $s=4\pi {\bar{R}}^{2}{\left(nm\right)}^{q}$ (*q* is the critical index). Therefore, with an independent specific flux *j* of the number of pores in the cluster, we obtain $\tau \left(m,n\right)=\tau_{0}m^{1-q}n^{q}$, $\tau_{0}=\frac{\bar{R}}{3}j^{-1}$. For the characteristic time $\tau_{pc}$ of the interaction of a cluster of *n* filled pores with a percolation cluster of the accessible pores, we similarly get $\tau_{pc}=\tau_{0}n^{-q^{\prime }+1}$. For $P<P_{0}$, the times $\tau_{0}$ and $\tau_{pc}$ characterize the rate of viscous flow and can be estimated by the dependence of the volume on time at the outflow stage (see Figure 2).

Equations (2) and (3) allow us to calculate the distribution functions of the clusters of accessible and filled pores by the number of pores in them for a given pressure change P(t). Equation (3) for f(n,t) contains terms whose physical sense is significantly different. The first three terms in the kinetic equation (3) do not have meaning of the collision integral, since they change only when ε=ε(t) and P(t). These terms are of the order of magnitude $\tau_{d}$ proportional to the time $\tau_{p}$, which is the internal time of the system, and reflect the change in the distribution function of accessible pores f(n,t) only when the fraction of accessible pores θ and, as a result, ε(t) change. If ε= const, then these terms are equal to zero. When ε=ε(t) they must be present in Equation (3) simultaneously with (∂f/∂ε)(dε/dt). Therefore, the derivative ∂f/∂t on the left-hand side of Equation (3), as well as the derivative ∂F/∂t determine the change in the function f(n,ε(t),t) and F(n,ε(t),t) due to a change in the rate of volume decrease during elastic deformation. This rate of change in the volume is controlled by external rapid compression and compressibility of the suspension. Solving Equations (2) and (3) with this in mind, one can obtain the distribution function and calculate the volume of fluid in the pores taking into account the response of the suspension to rapid compression.

Equations (2) and (3) contain the times corresponding to various processes that occur during elastic compression and filling of a porous medium: $\tau_{p}$ is the characteristic time of change in external pressure, $\tau_{d}$ is the characteristic time of formation of accessible pores, $\tau_{k}∼<\tau \left(n,m\right)>$ is characteristic formation time of filled pores cluster (angle brackets mean averaging over the ensemble of clusters of accessible and filled pores), $\tau_{\infty }∼<\tau_{\infty }\left(n\right)>$—characteristic time of fluid leaving into an infinite cluster of accessible empty pores, $\tau_{V}∼{\left(\partial \sum_{n=1}^{\infty }nF\left(n,t\right)/\partial t\right)}^{-1}$—the characteristic time of the change in the filled volume. For three-dimensional systems $\theta_{c0}=0.18$ and γ≈0.6, therefore, according to (4) and (5), always $\tau_{p}>\tau_{d}$. Since the filling of the volume occurs due to a change in external pressure, $\tau_{V}>$max$\left(\tau_{d},\tau_{k}\right)$.

As follows from the experiments, the stage of elastic compression of the suspension and the stage of filling the pores differ regarding the relative characteristic times of the processes. Estimates show that for a low relative compressibility of the suspension, $\tilde{\chi }∼10^{-2}$, the following relations hold: $\tau_{d}\ll \tau_{p}\ll \tau_{k}\ll \tau_{V}$. The distribution function of clusters of accessible pores can be formed on a time scale of $t∼\tau_{p}>\tau_{d}$. At $t∼t_{p}$, in the vicinity of $P_{c0}$, a percolation cluster of accessible pores can form. At pressure $P\geq P_{0}$ in the filling mode at P= const, the characteristic time $\tau_{p}>\tau_{V}$ and, consequently, the time hierarchy is different: $\tau_{d}\ll \tau_{k}\ll \tau_{V}∼\tau_{p}$. Therefore, the formation of clusters of accessible pores, the formation of a percolation cluster from accessible pores, the formation of a cluster of filled pores can be described by solving the system of Equations (2) and (3) sequentially on the scales of these characteristic times to the scale of $t\geq \tau_{V}$. Such a solution of Equations (2) and (3) is constructed below following the standard approach to multiscale problems solving in the physical kinetics [31]. The cluster distribution functions obtained on a smaller time scale, which describe faster processes, are used to solve the system of kinetic equations on the next, larger time scale, to describe slower processes.

In Equation (3) for the function f(n,t), the first term on the right-hand side is the main term, since it is of the order $\tau_{d}^{-1}$, while the second term is of the order $\tau_{k}^{-1}\ll \tau_{d}^{-1}$. Since $\tau_{p}<\tau_{k}$, a change in pressure at times $t∼\tau_{p}$ leads to the formation of accessible pores. At times $t<\tau_{k}$ there are no filled pores. Thus, for the times $t\geq \tau_{p}>\tau_{d}$ and $t<\tau_{k}$ taking into account $\frac{\partial \varepsilon }{\partial t}∼\tau_{d}^{-1}$ and the time dependencies τ(n,m) and $\tau_{pc}$ on the numbers *n*, *m* the system of Equations (2) and (3) has the form

(6) $$\begin{array}{c} F\left(n,t\right)\approx 0\\ \frac{\partial f}{\partial \varepsilon }=\frac{1}{2}\sum_{m=1}^{n-1}m^{q}{\left(n-m\right)}^{q}f\left(m,\varepsilon \right)f\left(n-m,\varepsilon \right)-\\ -n^{q}f\left(n,\varepsilon \right)\sum_{m=1}^{\infty }m^{q}f\left(m,\varepsilon \right)-\\ -2n^{q}f\left(n,\varepsilon \right)S\left(\varepsilon \right). \end{array}$$

Equation (6) has the form of the equation used in [22], the solution of which is known:

$$\begin{array}{c} f_{0}\left(n,t\right)=\frac{C\left(t\right)\Omega_{n}\left(t\right)}{Z\left(t\right)}, \end{array}$$

(7) $$\begin{array}{c} \Omega_{n}\left(t\right)=n^{-\tau }\exp\left[-r\varepsilon^{1/a}\left(t\right)n\right] \end{array}$$

(8) $$\begin{array}{c} Z\left(t\right)=\sum_{n=1}^{\infty }n\Omega_{n}\left(t\right). \end{array}$$

Here, the function C(t) is determined by the normalization of the distribution of $f_{0}\left(n,t\right)$. From (6) and (7) it follows that at times *t* satisfying the inequality $\tau_{V}>\tau_{k}>t\geq \tau_{p}>\tau_{d}$, the emerged accessible pores do not have time to fill with liquid, as a result of which the porous body is in a state above the percolation threshold $P_{c0}$ in accessible pores at $\theta >\theta_{c0}$ with F(n)≪f(n).

At times $\tau_{V}>t\geq \tau_{k}>\tau_{p}>\tau_{d}$, the process of filling the porous medium begins in accordance with Equations (2) and (3), where the effective part of the infinite cluster of accessible pores S(ε)≠0. At these times, due to the condition $t>\tau_{k}\gg \tau_{d}$, the time derivative in Equation (3) can be set equal to (dε/dt)(∂f/∂ε). This value is $∼1/\tau_{d}$ and, by virtue of the condition $\tau_{k}\gg \tau_{d}$, in the zero and first orders in $\tau_{d}/\tau_{k}$ in Equation (3) the sum of terms containing F(n,t) is zero. In this case, Equation (2) is satisfied automatically. Thus, at times *t* such that $\tau_{V}>t>\tau_{k}>\tau_{p}\gg \tau_{d}$, the equation for the distribution function of accessible pores f(n,t) coincides with the first equation in Equation (6), and the equation for F(n,t) takes the form

(9) $$\begin{array}{c} \left[\sum_{m=1}^{n-1}F\left(m\right)m^{q}{\left(n-m\right)}^{q-1}f\left(n-m,\varepsilon \right)-\right.\\ \left.F\left(n\right)n^{q-1}\sum_{m=1}^{\infty }m^{q-1}f\left(m,\varepsilon \right)\right]-\\ -F\left(n\right)n^{q^{\prime }-1}S\left(\varepsilon \right)=0. \end{array}$$

The equation for f(n,t) for S(ε)≠0 near $\theta_{c0}$ ($\theta \geq \theta_{c0}$) coincides with the one found in [22] and looks like (7). The function C(t), included in (7), determines the change in the volume of all accessible pores and changes at times $t∼\tau_{V}$, therefore, for $\tau_{V}>t>\tau_{k}\gg \tau_{d}$ it can be considered constant.

Equation (9) with the known distribution function of accessible pores (7) is a homogeneous equation for the function F(n). A nonzero solution to this equation exists only when the determinant of the matrix $A_{nm}$ vanishes:

(10) $$\begin{array}{c} \det A_{nm}=0,\\ A_{nm}=\Delta_{nm}{\left(n-m\right)}^{q-1}f_{0}\left(n-m,\varepsilon \right)m^{q}-\\ -\delta_{nm}\left[m^{q}\sum_{k=1}^{\infty }k^{q-1}f_{k}\left(k,\varepsilon \right)+m^{q^{\prime }-1}S\left(\varepsilon \right)\right],\\ \Delta_{nm}=\left\{\begin{array}{cc} 1 & n>m\\ 0 & n<m \end{array}\right. \end{array}$$

The matrix $A_{nm}$ has the form of a triangular matrix with zeros over the main diagonal. The determinant of such a matrix is equal to the product of diagonal elements,

(11) $$\det A_{nm}=\prod_{m}{\left(-1\right)}^{m}\left[m^{q}\sum_{k-1}^{\infty }k^{q-1}f_{0}\left(k,\varepsilon \right)+m^{q^{\prime }-1}S\left(\varepsilon \right)\right],$$

and does not vanish. Therefore, Equation (10) has no solutions for finite *n*, *m*. For n→∞, m→∞, the contact areas of two clusters are determined by one critical index, therefore $q\approx q^{\prime }-1$. Replacing summation in Equation (10) by integration, taking into account that $f_{0}{\left(n-m\right)|}_{n∼m}∼{\left(n-m\right)}^{-\tau }$ and setting

(12) $$\lim_{k\to 0}k^{q-1}f_{0}\left(k,\varepsilon \right)\approx 2\delta \left(k\right)\int_{0}^{\infty }dxx^{q-1}f_{0}\left(x,\varepsilon \right),$$

from (10) we find

(13) $$\begin{array}{c} \lim_{n,m\to \infty }A_{nm}=\lim_{n,m\to \infty }\delta_{nm}m^{q}\left[2\int_{0}^{\infty }dxx^{q-1}f_{0}\left(x,\varepsilon \right)-\right.\\ \left.-\int_{1}^{\infty }dxx^{q-1}f_{0}\left(x,\varepsilon \right)-S\left(\varepsilon \right)\right], \end{array}$$

where $\delta_{nm}$ is the Kronecker delta. From here follows an equation that determines the value of the fraction of accessible pores $\theta_{c}$, at which a nonzero distribution function of filled pores arises:

(14) $$2\int_{0}^{\infty }dxx^{q-1}f_{0}\left(x,\varepsilon \right)-\int_{1}^{\infty }dxx^{q-1}f_{0}\left(x,\varepsilon \right)-S\left(\varepsilon \right)=0.$$

From the expressions (7) and (14) for S(ε)≠0 it follows that the value $\theta_{c}$ is determined by the value of the percolation threshold $\theta_{c0}$ and the critical indexes included in (14). If the function $f_{0}\left(x,\varepsilon \right)$ is determined by Equations (7), then the integrals in (14) can be expressed in terms of the gamma function and Whittaker functions [32]. In this case, a numerical solution of Equation (14) for q=0.83, a=0.9 [22], $\theta_{c0}=0.18$ gives $\theta_{c}=0.28$.

Thus, Equation (9) has the solution F(n)=0 for $\theta_{c0}<\theta <\theta_{c}$ and F(n)≠0 for $\theta >\theta_{c}$. Therefore, we can say that at $\tau_{k}>\tau_{p}>\tau_{d}$ a new state of the system is formed for $\theta >\theta_{c}$. Further filling of the porous medium at times $t∼\tau_{k}$ can happen by its transition to the state that occurs in the case under consideration due to an infinite cluster of accessible pores. According to Equation (1) the pressure $P_{0}$ corresponding to the transition point of the porous medium to a new state is constant and is determined by the relation

(15) $$\int_{0}^{\infty }w\left(R,P_{c}\right)f_{r}\left(R\right)R^{3}dR=\theta_{c}.$$

From (15) the formula for *w* implies that the pressure $P_{c}$, in contrast to the value $\theta_{c}$, depends on the characteristics of the porous medium and liquid, such as, for example, the size distribution function of pores, correlations of neighboring pores, surface energies of a liquid and a porous medium.

Now we obtain an equation whose solution will allow us to analyze the stability of the state at $\theta >\theta_{c}$ under the conditions of formation of clusters of filled pores. Such an equation will also make it possible to find the time dependence of the volume fraction of pores filled with liquid at θ near $\theta_{c}$. Equation (3) can be represented as

(16) $$\frac{\partial F\left(n,\varepsilon ,t\right)}{\partial t}=\frac{1}{\tau_{0}\left(P\right)}\sum_{m=1}^{\infty }A_{nm}\left(\varepsilon \right)F\left(m,\varepsilon ,t\right).$$

The matrix $A_{nm}$ is defined by Equation (10), and its eigenvalues are given by the equation $\det(A_{nm}-\lambda \delta_{nm})=0$. For finite values of *n* and *m* we have

(17) $$\det\left(A_{nm}-\lambda \delta_{nm}\right)=\prod_{m}\left[-\lambda -\left[m^{q}\sum_{k=1}^{\infty }k^{q-1}f_{0}\left(k,\varepsilon \right)+m^{q^{\prime }-1}S\left(\varepsilon \right)\right]\right].$$

and therefore, for finite *n*, *m*, the eigenvalues of the matrix $A_{nm}$ are negative. For n→∞, m→∞, according to (13), we get

(18) $$\begin{array}{c} \lambda =\lambda_{\infty }\left(\theta \right)\approx \langle m^{q}\rangle \left[2\int_{0}^{\infty }dxx^{q-1}f_{0}\left(x,\varepsilon \right)-\right.\\ \left.-\int_{1}^{\infty }dxx^{q-1}f_{0}\left(x,\varepsilon \right)-S\left(\varepsilon \right)\right]=z{\left(\theta -\theta_{c}\right)}^{\zeta }. \end{array}$$

Angle brackets correspond to averaging over an ensemble of clusters at m≫1; *z*, ζ are constants. Numerical calculations at q=0.83, a=1, δ=0.2 give z≈0.8, ζ≈0.8. Thus, in the spectrum of eigenvalues of the matrix $A_{nm}$ for n→∞, m→∞ and the fraction of accessible pores $\theta \geq \theta_{c}$, a small positive eigenvalue appears corresponding to relaxation time $\tau_{\infty }∼{\left(\theta -\theta_{c}\right)}^{-\zeta }\tau_{k}$, while other eigenvalues are finite at $\theta =\theta_{c}$, are negative and are of the order $\tau_{k}^{-1}$.

Using Equation (18), we rewrite Equation (16) in the form

(19) $$\frac{\partial F\left(n\right)}{\partial t}=\frac{\lambda_{\infty }\left(\theta \right)}{\tau_{0}\left(P\right)}F\left(n\right)+\sum_{m}{\tilde{A}}_{nm}F\left(m\right).$$

The matrix

(20) $${\tilde{A}}_{nm}=\frac{1}{\tau_{0}\left(P\right)}A_{nm}-\frac{\lambda_{\infty }\left(\theta \right)}{\tau_{0}\left(P\right)}\delta_{nm}$$

has negative eigenvalues λ(n)<0, $\left|\lambda \left(n\right)\right|∼1/\tau_{k}$ that do not vanish at $\theta =\theta_{c}$. The time derivative in Equation (19), should describe the change in *F* at two time scales—the scale of relaxation of fluctuations in the formation of clusters of filled pores $t∼{\left|\lambda \left(n\right)\right|}^{-1}∼\tau_{k}$ and the time scale $t∼\tau_{\infty }∼\tau_{k}{\left(\theta -\theta_{c}\right)}^{-1}$ of exponential increase in filling.

In general, the distribution function of clusters of filled pores can depend on time additionally through pressure, since pressure determines the fraction of accessible pores and hence in Equations (2) and (3). At the time ratio $t∼\tau_{\infty }<\tau_{V}\ll \tau_{p}$ and, therefore, when the growth rate of the unstable mode is greater than the pressure change rate, the small term describing the time dependence of the function *F* through pressure $P\left(t\right)$ can be neglected in Equation (1). Then we can take $\dot{P}=0$ and the equation describing the dependence of the amplitude $A\left(t\right)$ of the unstable mode has the form:

(21) $$\frac{dA\left(t\right)}{dt}=\frac{A\left(t\right)\theta_{0}}{\tau_{\infty }}.$$

According to this equation, the rate of transport and the degree of filling of the liquid must be described by an exponentially increasing dependence on time. Earlier in [13], the equation $\dot{A}∼A\left(1-A\right)$ was derived for the opposite case of small pressure rise time, when $\tau_{p}\ll \tau_{V}$. It follows (21) that the filling rate is determined by the product of the fraction of filled pores $A\left(t\right)$ and the fraction of accessible pores in the percolation cluster and the characteristic time $\tau_{\infty }$ of the unstable mode, which matches that found in [13].

The high frequencies $\omega_{k}∼\tau_{0}^{-1}$ are negative and depend on the number of filled pores in the clusters. These are the frequencies of the spectrum of the relaxation process of intrusion-extrusion liquid from clusters containing different finite number of filled pores. In accordance with the method of exclusion of fast modes [33,34,35] in Equation (21) the term corresponding to high-frequency intrusion-extrusion processes, which would turn this equation into a stochastic one, is removed. This corresponds to the adiabatic approximation [34]. The positive frequency $\omega_{\infty }=\tau_{\infty }^{-1}$ of the unstable evolution mode in the percolation limit of large particle sizes L≫R (*R* is the average pore radius) is determined by the expression:

(22) $$\tau_{\infty }=\frac{\tau_{0}}{z{\left(\theta -\theta_{c}\right)}^{\zeta }},$$

where z≈1, ζ=0.8, θ is the pressure-dependent fraction of accessible pores. In the formula (22) $\theta_{c}$ is the critical fraction of accessible pores of the dynamic percolation transition. The value $\theta_{c}=\theta_{c0}+0.1$ calculated in [13], $\theta_{c0}=0.18$ is the critical fraction of accessible pores in the quasistatic regime. When the fraction of accessible pores $\theta <\theta_{c}$, according to (22), intrusion should not be observed. As the pressure increases and the fraction of accessible pores increases accordingly, the time for evolution of the instability decreases from $\tau_{\infty }∼\infty$ at $\theta =\theta_{c}$ to a minimum scale $\tau_{0}$ at $\theta =\theta_{0}$. As a result, the rate of evolution of an unstable mode increases with increasing pressure from $P_{c}$ to $P_{0}$ and with a delay reaches the maximum value at pressure $P_{0}$. Then the rate of evolution of an unstable mode becomes close to the rate of filling $\tau_{0}^{-1}$ of a cluster of the finite size. On a time scale $t∼\tau_{\infty }\ll \tau_{p}$ the pressure growth rate $\dot{P}=0$ and at constant pressure $P_{0}$ the fraction of accessible pores is constant $\theta =\theta_{0}$ regardless of the size of the pore volume filled by the liquid.

At impact compression under the constant pressure regime, the change of suspension volume occurs due to pore-filling without elastic compression. Pore-filling in this mode is the result of two different processes: spontaneous filling and filling caused by pore volume reduction during impact compression of the suspension in the volume of the chamber fully filled with the suspension. To take into account the change in the fraction *x* of filled pores during shock compression when deriving the equation for $x\left(t\right)$ it is necessary to consider the dependence of the function $F\left(k,t,x\left(t\right)\right)$ on the rate of change of the macro parameter $x\left(t\right)$. This allows us to describe the change in the distribution function over a larger time scale than $\tau_{\infty }$, $\tau_{V}\tau_{\infty }$, $\tau_{V}\ll \tau_{P}$.

In the experiments [10,11,13,27] the impact compression of the suspension was studied on an impact stand. A load of mass *M* was dropped on a rod that entered a chamber filled with the suspension. In such a technique, at P=const, the volume of the suspension is reduced only by filling the pores. Then we can obtain an expression for the rate of change in the fraction of filled pores during impact compression in the form:

$$\frac{dx}{dt}=\frac{v_{r}\left(t\right)S}{V_{0}},$$

(23) $${\dot{v}}_{r}=-\frac{PS}{M},$$

$$v_{r}\left(t\right)=v_{r}\left(0\right)-\frac{PS}{M}t,$$

where $v_{r}\left(t\right)$ is the velocity of the rod with a mass of *M*, *S* is the area of the rod, $V_{0}$ is the pore volume of the suspension particles.

It follows from (23) that the transport rate due to impact compression decreases with time due to the braking of the load with acceleration *a* at constant pressure $P_{0}$. The solutions (21) and (23) are obtained for the distribution function of clusters of filled pores as product $F\left(k,t\right)=x\left(t\right)A\left(t\right)F\left(k\right)$. According to Equation (22), the value $A\left(t\right)$ changes on a small time scale $\tau_{\infty }$. The value $x\left(t\right)$ determines the time dependence of the fraction of filled pores on a larger time scale $\tau_{V}$, $\tau_{V}\gg \tau_{\infty }$. The value of the transport rate should be determined by the two processes occurring simultaneously at two different time scales. These times can be close, according to (21) and (23) at x≪1 and differ by an order of magnitude at x∼1. Therefore, to describe the nonstationary transport it is necessary to take into account the interaction of modes. In experiments [10,11,13,27] such interaction follows from the condition of keeping the hydraulic contact of the rod with the suspension. Let us assume that the characteristic time of the evolution of the fast mode of spontaneous filling $\tau_{\infty }$ is much less than the characteristic time of filling during impact compression $\tau_{V}$. Then equality of rates of volume change can be written as equality, for time $\tau_{\infty }$, increment of filling Δx caused by spontaneous filling mode and increment of filling rate caused by the impact compression:

(24) $$\frac{\Delta x\theta_{0}}{\tau_{\infty }}=\frac{\Delta v_{r}S}{V_{0}}=\frac{PS}{M}\frac{S}{V_{0}}\tau_{\infty }.$$

According to (24) on the time interval from 0 to $\tau_{\infty }$ the filling process is described by Equation (21) and is a spontaneous filling process. Over a long time interval $\tau_{V}$ the filling process is described by Equation (23) for the impact mode of compression. The relation (24) represents the initial and final boundary conditions for Equation (21) and determines the change of filling at each small interval $\tau_{\infty }$. The change in the fraction of filled pores over time $\tau_{\infty }$ must decrease as the rate of filling during the impact compression decreases. Thus, fast spontaneous filling follows slow impact compression, adjusting to sequences of small-scale time intervals $\tau_{\infty }$. The time dependencies of liquid transport rate, filled pore volume, and pressure observed in the experiments should be described by a slow impact compression mode and Equation (23).

According to (23) the flow rate in the experiments should depend linearly on time. Then the filled pore volume, according to (23) and (24) should be a quadratic function of time on the interval up to the maximum pore-filling. This means that transport at P=const is effectively non-dissipative, which corresponds to the observed [13,27] invariance of the volume and pressure dependencies on time as the temperature and viscosity of the liquid change.

## 5. Comparison with the Experiment

**Figure 3:**
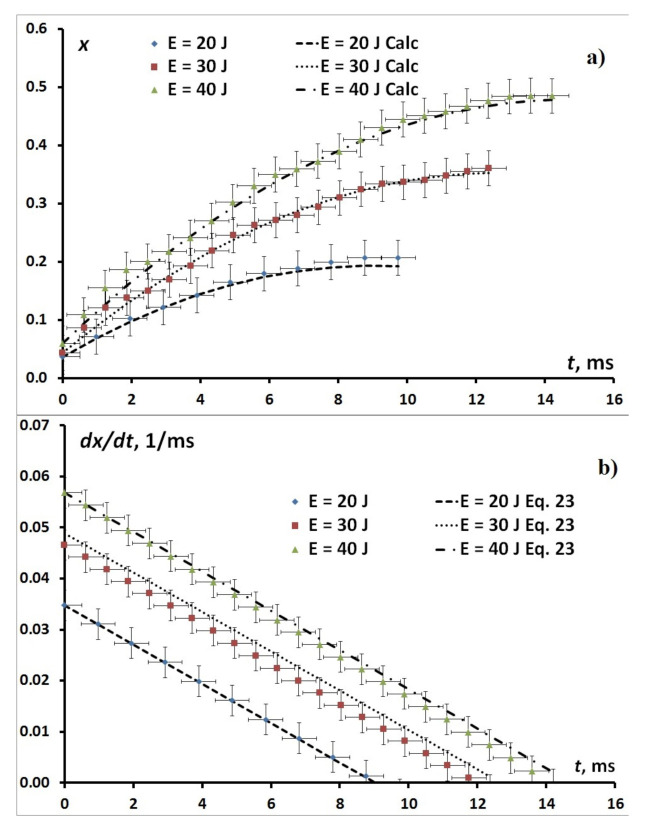
The experimental time dependencies of (**a**) the fracture of filled pore volume $x\left(t\right)$ and calculated using the Formula (23) and (**b**) normalized flux with calculations in accordance with the (23). The time is counted from the time $t_{2}$ reaches the pressure $P_{0}$.

Figure 3 shows the time dependencies of the fracture of filled pore volume $x\left(t\right)$ and derivative of the fracture of filled pore volume $dx\left(t\right)/dt$ (normalized flux) for the time interval from reaching pressure $P_{0}$ (see Figure 1), calculated by the Formula (23), and experimental data. The time in Figure 3 is counted from the time $t_{2}$ reaches the pressure $P_{0}$. The time of the end of the dependencies corresponds to the time $t_{3}$ of Figure 1. This point corresponds to the moment of complete absorption of the impact momentum. The curves are given for three values of energies E= 20, 30, 40 J. It can be seen that as the energy increases, the pore-filling end time and the time to reach zero pore-filling flux increase. In the energy interval of 20÷40 J the filling occurs at constant pressure and, according to the formula (23), there is a linear dependence of derivative of the fracture of filled pore volume $dx\left(t\right)/dt$. As the impact energy increases, the initial normalized flux and the value of the fracture of filled pore volume before the process at P=const increases.

It can be seen that within the measurement errors, the experimental data are described by theoretical dependencies. This corresponds to the description of transport in the system as a multiscale process. In this multiscale process, the characteristic growth time of the spontaneous filling (fast mode) can be estimated from the time interval $t_{1}÷t_{2}$ of the duration of the transient process from elastic compression of the suspension to filling at constant pressure $P_{0}$. The critical pressure of dynamic transition $P_{c}$ lies between pressures $P_{0}$ and $P_{c0}$. According to the Formula (22), the filling process can begin at pressures $P_{c}>P_{c0}$, $P_{c}<P_{0}$. Therefore, we can assume that $\tau_{\infty }\approx \frac{1}{2}\left(t_{2}-t_{1}\right)$. For energies 20, 30, 40 J, the value of $\tau_{\infty }\approx 1$ ms.

The characteristic filling time $\tau_{V}$ is equal to the difference between the times of reaching zero flux and the beginning of filling $\tau_{V}=t_{3}-t_{2}$. For energies 20, 30, 40 J the value of $\tau_{V}$ varies from 10 ms to 15 ms. The relation between the characteristic times of the slow mode – filling caused by impact compression at pressure $P_{0}$ – and spontaneous filling is $\tau_{V}/\tau_{\infty }=10÷15$. Thus, $\tau_{V}\gg \tau_{\infty }$.

Now let us discuss the description of the experimental data within the framework of the multiscale model of interaction of modes. According to this model, the observed dependencies should be described by the slow mode as a filling mode caused by impact compression, which is confirmed by the dependencies given in Figure 3. The fast mode (spontaneous filling), according to the model, follows the slow mode at each of the time intervals $\tau_{\infty }$, the number of which is determined by the ratio $\tau_{V}/\tau_{\infty }$. Thus, the filling process is determined by the slow mode and is followed by continuous adjustment of the fast mode to the slow mode on the scale $\tau_{\infty }$.

Equation (21) does not contain the dependence of flow $J=V_{0}\frac{dx}{dt}$ on pressure. In addition, the time $\tau_{\infty }$ in (21) and (22) is proportional to ${\left(\theta_{0}-\theta_{c}\right)}^{\zeta }$ and is a constant minimum time of instability evolution when $P=P_{0}=const$. The liquid transport described by Equation (21) is spontaneous. This result corresponds to the kinetic theory, which takes into account in the system of initial kinetic equations for cluster distribution functions the “interaction” of clusters of accessible pores and clusters of filled pores. The probability of this “interaction” is inversely proportional to the time of filling of the accessible cluster with liquid. Since “interaction“ means overflow, the result is the formation of new clusters of filled pores. The growth of the filled volume is described as a result of the “interaction” of clusters from the filled pores with the percolation cluster of accessible pores – liquid flow from the particle surface through the clusters of filled pores into the percolation cluster of accessible pores. The characteristic time (22) of exponential growth of the growing mode is independent of the number of filled pores in such clusters. Therefore, “interaction” means the filling of the percolation cluster of accessible pores, and thus of the entire pore system simultaneously through all different clusters of filled pores. Such spontaneous transport can be considered to be cooperative.

**Figure 4:**
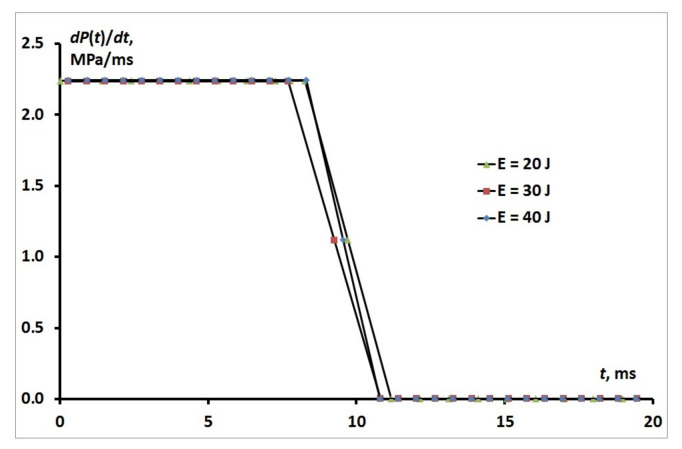
Transition of the $\dot{P}$ from elastic compression of suspension to filling.

The liquid transport at $t∼\tau_{\infty }\ll \tau_{P}$ is described in the approximation $\dot{P}=0$ and hence $P=P_{0}=const$. The dimensionless rate of pressure growth $\dot{\tilde{P}}$ is related, in the hydrodynamic limit, to the dimensionless rate $\tilde{J}$ of transport and the dimensionless compressibility $\tilde{\chi }$, $\tilde{J}=\tilde{\chi }\dot{\tilde{P}}$. Then, given a finite value of $\tilde{J}$, and $\dot{\tilde{P}}\to 0$ it follows that $\tilde{\chi }\to \infty$. In the experiments, the effective dimensionless compressibility of the suspension without pore-filling was determined by the compressibility of the liquid and for water ${\tilde{\chi }}_{el}∼10^{-2}$. As a result, in the process of pressure growth in the vicinity of the critical value $P=P_{c}$ and transition from elastic compression of suspension at ${\tilde{\chi }}_{el}∼10^{-2}$ to filling at the small time interval $t∼\tau_{0}$, a jump $\dot{P}$ is observed (see Figure 4).

The observed dependencies are obtained at $P_{0}=const$, and the value of $P_{0}$ does not depend on the impact energy and liquid flow in the pores. This means, as noted above, that transport at P=const is effectively non-dissipative. The dissipationless transport is consistent with the invariance of the time dependencies of the filled volume and pressure with changes in temperature and liquid viscosity obtained in works [10,11,13].

According to (21)–(24), spontaneous transport follows a time scale $\tau_{\infty }$ following rapid impact compression. As the impact compression energy changes (decreases) over time $\tau_{\infty }$, the fraction of filled pores changes and, according to (21), the rate of spontaneous transport changes. Therefore, it can be said that the rate of spontaneous transport ”adjusts“ to changes in the rate of external influence. With this property, the spontaneous transport changes in such a way that it contributes to the change in the external action. This corresponds to a response with the positive feedback property.

## 6. Conclusions

It follows from the foregoing that with rapid compression, a multiscale process occurs, which can be described as the formation and decay of an unstable state of a suspension of particles with empty pores. The key to the formation of an unstable state is elastic compression. The characteristic pressure increase time $\tau_{p}$ in this process is much shorter than the characteristic time of filling the percolation cluster of accessible pores at $P∼P_{c}$ and the time of filling $\tau_{0}$ of individual clusters of accessible pores at a pressure in the vicinity of $P_{0}>P_{c}$. Therefore, due to the filling delay, the system of particle pores turns out to be unfilled in the vicinity of the critical point $P_{c}$. The critical pressure $P_{c}$ is independent, in accordance with (23), of the compression energy and particle mass and is a property of a disordered system of pores and a non-wetting liquid. The mode of pore-filling at a constant liquid transport rate can be kept if, with increasing impact energy, the mass of particles and, consequently, the volume of accessible pores increases. The barrier of the fluctuation filling of pores in the vicinity of $P_{0}$ for the studied suspension is comparable to the temperature for pores of the minimum size ($R_{min}$) in the distribution of f(R). For larger pores, the barrier is lower. As a result, at $P∼P_{0}$, fluctuations in the filling of pores with $R<R_{min}$ are unstable, and during the time the pressure rises to $P_{0}$, almost all pores of particles are accessible to a non-wetting liquid.

However, it turned out that filling is not related to the evolution of such fluctuations, and the condition of thermodynamic instability is only a necessary condition at the critical point $P_{0}$. At this point, the system of empty accessible pores of particles in a non-wetting liquid transforms into a dynamic state with a developing collective mode of "interaction" of an ensemble of clusters of filled pores with a percolation cluster of accessible pores. Such an “interaction” leads to an exponential acceleration of filling. In accordance with the model of the interacting modes, it is possible to control filling by a slow mode. The control parameter is the rate of change of volume, $\dot{V}=\tau_{V}^{-1}=v_{r}SV^{-1}$ (*S* and $v_{r}$ are the area and velocity of the rod) of the suspension, associated with the “effective” compressibility χ when filling at the pressure increase rate, $\dot{P}=\tau_{p}^{-1}$, $\dot{V}=\chi \dot{P}$ and if P=const, $\dot{P}=0$, χ→∞ at $t_{2}÷t_{3}$. Thus, the considered theoretical model based on the solution of the kinetic equations for the distribution functions of clusters of filled and accessible pores, and the equations obtained from the solution for the macroscopic values of the amplitude of the unstable fast mode and the fraction of the filled pore volume, allow us to describe the experimental data. The developed model allows us to remove the contradictions arising from the observed dependencies of the flow and volume of filled pores on time as caused by impact compression, but occurring at P=const, which indicates the spontaneous non-dissipative nature of the transport. The evidence of the influence of two interacting modes on the transport process is the unusual response of the suspension with positive feedback to the external interaction.
